# Effects of nanoparticles of hydroxy-aluminum phthalocyanine on markers of liver injury and glucose metabolism in diabetic mice

**DOI:** 10.1590/1414-431X20187715

**Published:** 2018-12-03

**Authors:** M.A.B. Melo, W. Caetano, E.L. Oliveira, P.M. Barbosa, A.L.B. Rando, M.M.D. Pedrosa, V.A.F. Godoi

**Affiliations:** 1 Programa de Pós-Graduação em Ciências Fisiológicas, Departamento de Ciências Fisiológicas, Universidade Estadual de Maringá, Maringá, PR, Brasil; 2 Departamento de Química, Universidade Estadual de Maringá, Maringá, PR, Brasil; 3 Departamento de Biologia, Universidade Estadual de Maringá, Maringá, PR, Brasil; 4 Departamento de Ciências Fisiológicas, Universidade Estadual de Maringá, Maringá, PR, Brasil

**Keywords:** Hydroxy-aluminum phthalocyanine, Hydrogel, Liver, Type 1 diabetes, Mice

## Abstract

Photodynamic therapy, by reducing pain and inflammation and promoting the proliferation of healthy cells, can be used to treat recurrent lesions, such as diabetic foot ulcers. Studies using the photosensitizer phthalocyanine, together with the nanostructured copolymeric matrix of Pluronic^®^ and Carbopol^®^ for the treatment of diabetic foot ulcers and leishmaniosis lesions, are showing promising outcomes. Despite their topical or subcutaneous administration, these molecules are absorbed and their systemic effects are unknown. Therefore, we investigated the effect of the subcutaneous administration of the hydroxy-aluminum phthalocyanine hydrogel without illumination on systemic parameters, markers of liver injury, and liver energy metabolism in type 1 diabetic Swiss mice. Both the hydrogel and the different doses of phthalocyanine changed the levels of injury markers and the liver glucose release, sometimes aggravating the alterations caused by the diabetic condition itself. However, the dose of 2.23 µg/mL caused less marked plasmatic and metabolic changes and did not change glucose tolerance or insulin sensitivity of the diabetic mice. These results are indicative that the use of hydroxy-aluminum phthalocyanine hydrogel for the treatment of cutaneous ulcers in diabetic patients is systemically safe.

## Introduction

Diabetes mellitus, a worldwide health problem characterized mainly by chronic hyperglycemia, is a disease affecting 450 million people around the world (1). Among its chronic complications, the distal symmetric diabetic polyneuropathy, which afflicts the peripheral nervous system, is responsible for 75% of the neuropathies in these patients (2).

The diabetic foot, a classical neuropathic complication of type 1 diabetes mellitus (T1DM), is characterized by plantar ulcers of slow healing that may become chronic (3,4). It accounts for 84% of leg amputations (5). Several factors are reported as relevant for the impaired healing, such as excessive production of reactive oxygen species (ROS), decreased nitric oxide, diminished response to growth factors, and reduced activation of the insulin signaling pathway (6).

Among the different methods for the treatment of the diabetic foot, photodynamic therapy (PDT) can cause healing (7) and decrease pain and inflammation by causing the proliferation of healthy cells, stimulating collagen synthesis, and increasing the immunity and the mechanical resistance of the lesion (7). PDT uses a photosensitizer (PS), a specific wavelength, and molecular oxygen (8). When irradiated with light, the PS generates cytotoxic species from molecular oxygen, such as singlet oxygen (^1^O_2_) and ROS, which cause the death of the injured tissue (9,10).

Phthalocyanines are PSs derived from synthetic porphyrins of high quantic yield and absorption in the red electromagnetic region (600-800 nm), which corresponds to the therapeutic range of PDT (11,12); for instance, tetrasulphonate aluminum phthalocyanine is the active principle of Photosens^®^ (State Research Center of Organic Intermediates and Dyes, Russia) used in the treatment of some types of cancer (13). As hydrophobic photoactive drugs, phthalocyanines interact easily with cell membranes (14), but demand solubilizer/transporter systems (15).

The micellar triblock copolymers of the Pluronic^®^ (BASF, USA) class are widely used drug carrier systems that are biocompatible, biodegradable, non-ionic, and thermo-responsive (16). These properties of Pluronic^®^ can be combined with the adhesive properties of Carbopol^®^ (Sakshi, India), a highly hydrophilic polymer used in the pharmaceutical industry because of its muco-adhesive properties (17). The copolymeric matrix of Pluronic^®^ F-127, Carbopol^®^, and photoactive drugs maintains the viscous and rheological properties of the hydrogels, enabling the encapsulation of the PS and its release after application to the skin. This type of formulation can promote the photo-stimulated elimination of microorganisms (bacteria, fungi, virus, and protozoans) that cause dermatitis, onychomycoses, and other skin and mucosal infections; it is also used against different types of tumors (11,12,18).

The use of PDT for the treatment of diabetic ulcers has been successful (7). Similarly, the topical or subcutaneous application of PS with the nanostructured copolymeric matrix of Pluronic^®^ and Carbopol^®^ for the treatment of diabetes and leishmaniosis ulcers has shown promising results (Caetano W, data not published). However, these molecules are absorbed by the systemic circulation, especially because the PS, despite being hydrophobic, is transported by a hydrophilic gel. When it reaches the organs, the PS could have adverse effects, especially in the liver and kidneys, responsible for the detoxification and excretion of xenobiotics and that are compromised by long-term diabetes. Assays of the biodistribution of intravenous phthalocyanine showed a greater accumulation in the liver, followed by cancer tissue, pointing to the liver as a central organ in the uptake of these molecules (19,20).

The effects of the phthalocyanines and the hydrogel at the systemic level and on liver energy metabolism in experimental animal models are not known but could be relevant both physiologically and clinically. This investigation tested the systemic and hepatic effects of three different concentrations of the PS hydroxy-aluminum phthalocyanine and the vehicle used in creams and ointments, the hydrogel of Pluronic^®^+Carbopol^®^, without illumination. The hypothetical basis was that the PS as well as the vehicle could be absorbed and taken up by the tissues, where they could exert light-independent actions. Swiss mice with T1DM induced by alloxan and not insulin-treated, hyperglycemic, and metabolically decompensated were used.

## Material and Methods

### Preparation and use of the Pluronic^®^+Carbopol^®^+phthalocyanine hydrogels

**Figure 1:**
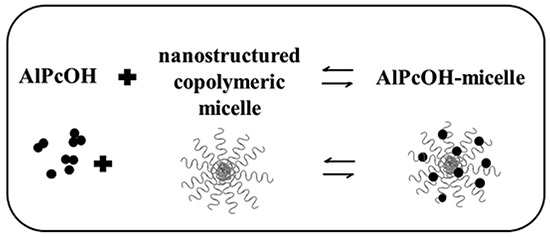
Schematic representation of the encapsulation of phthalocyanine AIPcOH into the Pluronic^®^ F-127 micelle.

Hydroxy-aluminum phthalocyanine was encapsulated in copolymeric micelles of Pluronic^®^ F-127 (20%), with further dispersion in a hydrated solution containing Carbopol^®^ 934P (0.2%). A schematic representation of the micelle is shown in Figure 1. The phthalocyanine doses of 2.23, 250, and 560 µg/mL, and the subcutaneous injection were determined by the Research Group in Photodynamic Systems (NUPESF) from the State University of Maringá, responsible for the development and production of the compound, based on published work (21,22) and on previous *in vitro* investigations of that research group (11,12,23). The injection volume of 100 µL as a single dose was chosen after preliminary tests on mice to verify the reaction of the injected site to different volumes and schedules of injection (data not shown).

The thermo-responsive hydrogel (vehicle) promoting the nanoencapsulation of the phthalocyanine into the nanostructured system (copolymeric micelle of Pluronic^®^ F-127+Carbopol^®^ 934P) undergoes complete degradation in 14 days at 37°C, but *in vivo* degradation can be faster (24), resulting in the release of phthalocyanine. Phthalocyanine is released as the hydrogel is degraded and remains free for 48 h before being completely excreted. Therefore, preliminary experiments demonstrated that the subcutaneous single injection of 100 µL of the hydrogel either alone or containing the phthalocyanine, with *in situ* liver perfusion on the sixth day after injection, allowed good assessment of their effects on liver metabolism.

### Experimental groups

Adult male Swiss mice (40 days old, 30 g body weight) were kept at the animal house under controlled temperature (23-25°C) and photoperiod (12 h light/12 h dark). The animals were given standard rodent chow (Nuvital^®^, Nuvilab, Brazil) and water *ad libitum*. All procedures were approved by the Ethics Commission on Animal Use of the State University of Maringá (CEUA 4191310117).

After overnight fasting (15 h), some of the animals were injected *ip* with alloxan (Sigma, USA) at the dose of 180 mg/kg body weight in 10 mM citrate buffer, pH 4.5. After seven days, animals with post-prandial and fasting blood glucose ≥300 mg/dL were considered diabetic (GD). Non-diabetic animals (control group, GC) were injected *ip* only with citrate buffer.

The GD mice were subdivided according to treatment as follows: GD-G: diabetic animals that were injected with hydrogel only (20% F-127 + 0.20% C934P); GD-F2.23: diabetic animals that were injected with hydrogel + 2.23 µg/mL phthalocyanine (F2.23); GD-F250: diabetic animals that were injected with hydrogel + 250 µg/mL phthalocyanine (F250); GD-F560: diabetic animals that were injected with hydrogel + 560 µg/mL phthalocyanine (F560).

### Subcutaneous administration of the compounds

The hydrogel alone (group GD-G) or hydrogel+phthalocyanine (groups GD-F2.23, GD-F250, and GD-F560) were injected as a single dose, into the subcutaneous tissue of the dorsal neck, at the volume of 100 µL, on the fifth day after confirmation of the diabetic state.

All the *in vivo* and *in vitro* experiments were carried out in the morning, after overnight fasting (15 h), six days after the subcutaneous injection. Illumination was not carried out during this period.

### *In situ* liver perfusion

The animals were anesthetized (40 mg/kg thiopental + 5 mg/kg lidocaine, *ip*, 0.6 mL/100 g body weight) and had the liver perfused (4 mL/min per g of liver) with glucose-free Krebs/Henseleit-bicarbonate (KH) buffer, pH 7.4, aerated (O_2_:CO_2_, 95%:5%) and warmed (37°C) in a non-recirculating system. The buffer influx was through the portal vein and the efflux through the inferior cava vein, so that the natural anterograde direction of the hepatic lobular circulation was preserved. Euthanasia occurred through hypovolemic shock.

Perfusate samples were collected every five min for 60 min for further biochemical analyses. The initial 30 min were considered basal period (perfusion with KH only) and the next 30 min stimulated perfusion, during which the gluconeogenic precursors glycerol (4 mM) and lactate (4 mM) were added to the KH buffer.

### Blood collection and biochemical assays

The effluent fluid collected during perfusion was used to determine the concentration of glucose and pyruvate, expressed as µmol/min per g of liver. The area under curve (AUC) of liver glucose and pyruvate release (in µmol/g liver) was calculated for the basal and stimulated periods of the perfusion.

Blood was collected immediately after the animals were anesthetized for perfusion through cardiac puncture. Assessment of liver injury was made by determining the plasmatic concentration of aspartate amino transferase (AST), alanine amino transferase (ALT), alkaline phosphatase (ALP), and gamma-glutamyl transferase (GGT). Additionally, fructosamine (FRU), total cholesterol (CHOL), triglycerides (TGL), and glucose (GLU) were measured. Laboratory kits (Gold Analisa^®^ Diagnóstica Ltda, Brazil) were used following the specifications of the supplier.

### Determination of liver glycogen

The dose of hydroxy-aluminum phthalocyanine that had less effects on liver glucose metabolism and on plasmatic markers of liver injury was selected to evaluate liver glycogen content. For comparison, liver glycogen of groups GC and GD was also determined.

Livers were quickly removed, immersed in liquid nitrogen and weighed. Part of the organ was ground, perchloric acid (0.6 N) was added, and the resulting mass was homogenized and centrifuged (4,829.76 *g* at room temperature for 10 min). Aliquots of the supernatant were used to determine the levels of free glucose (Gold Analisa^®^, Brazil). Amyloglucosidase was added to another aliquot of supernatant, along with potassium bicarbonate (1 M) and sodium acetate (250 mM). The solution was incubated at 40°C in a water bath under agitation for 2 h, and the enzymatic reaction was interrupted by the addition of perchloric acid. Finally, after another centrifugation (4,829.76 *g* at room temperature for 10 min), aliquots of supernatant were used to determine total glucose concentration, i.e., free glucose plus glycogen-derived glucose (Gold Analisa^®^). Data are reported as mmol glucose/g liver.

### *In vivo* experiments

The dose of hydroxy-aluminum phthalocyanine that had less effects on liver glucose metabolism and on plasmatic markers of liver injury was used for *in vivo* glucose tolerance test (GTT) and insulin sensitivity test (ITT).

The mice of groups GC, GD, and GD-F were fasted overnight (15 h). For the GTT, the animals were given oral glucose solution (1.5 g/kg body weight) and blood was collected form the caudal vein at times 0 (immediately before oral glucose), and 5, 15, 30, 45, and 60 min after. Blood glucose was measured with test strips and glucometer (Optium Exceed^®^; Abbott, Brazil). The AUC of blood glucose variation during the 60 min was calculated taking basal blood glucose (time 0 min) of each animal as baseline. At the end of the GTT, the animals were returned to their cages and fed.

Forty-eight hours later, the animals, after 6 h of fasting, were given an *ip* injection of insulin (1 U/kg body weight; Novolin^®^, Novo Nordisk, Brazil) and blood from the caudal vein was collected at times 0 (immediately before insulin injection), and 5, 10, 15, 20, and 30 min after insulin injection. Blood glucose was determined with test strips and glucometer (Optium Exceed^®^). Based on these data, the blood glucose decay index (kITT, in %/min) was calculated for the 30 min of the test.

### Statistical analyses

The data are reported as mean±SD and were subjected to Shapiro-Wilk normality test. To characterize the diabetic model, the control (GC) and diabetic (GD) groups were compared through *t*-test. The diabetic groups (GD, GD-G, GD-F) were compared using one-way ANOVA with Tukey’s *post hoc* test. The significance level adopted was pre-fixed at 95% (P<0.05). Statistical analyses and graphs were made using Prism^®^ 5.0 (GraphPad, USA).

## Results

### Liver perfusion

Table 1 shows the AUC values of liver glucose release (LGR) and liver pyruvate release (LPR) during the basal and stimulated periods of perfusion. Basal LGR was higher in GD compared to GC, while groups GD-G and GD-F560 had higher basal LGR compared to group GD. Of the three doses of phthalocyanine, the one with highest basal LGR was F560. In addition, the phthalocyanine at the lowest dose (F2.23) decreased basal LGR to the level of group GD.

**Table 1:** Liver glucose and pyruvate release of mice from groups GC, GD, GD-G, GD-F2.23, GD-F250, and GD-F560.

AUC (µmol/g liver)	GC	GD	GD-G	GD-F2.23	GD-F250	GD-F560
bLGR	2.70±0.72	7.95±2.47a	12.43±0.75b	7.57±1,79c	10.25±2.97	19.08±1.32bcd
sLGR	16.78±0.67	10.36±1.55a	8.63±1.73	3.72±1.67bc	6.38±2.19b	6.65±2.58b
bLPR	0.083±0.024	0.049±0.026	0.143±0.038b	0.126±0.011b	0.023±0.001cd	0.070±0.018c
sLPR	1.595±0.328	2.633±0.377a	2.896±0.648	1.128±0.602bc	1.506±0.197bc	1.090±0.525bc

GC: control group; GD: diabetic animals; GD-G: diabetic animals that were injected with hydrogel only; GD-F2.23: diabetic animals that were injected with hydrogel + 2.23 µg/mL phthalocyanine; GD-F250: diabetic animals that were injected with hydrogel + 250 µg/mL phthalocyanine; GD-F560: diabetic animals that were injected with hydrogel + 560 µg/mL phthalocyanine. AUC: area under curve; bLGR: basal liver glucose release; sLGR: stimulated liver glucose release; bLPR: basal liver pyruvate release; sLPR: stimulated liver pyruvate release; Data are reported as means±SD; n=4-6/group. a: P<0.05 *vs* GC (*t*-test); b: P<0.05 *vs* GD; c: *vs* GD-G; d: P<0.05 *vs* GD-F2.23 (one-way ANOVA/Tukey).

During gluconeogenesis stimulation, LGR was lower in GD compared to GC. The groups that received phthalocyanines (GD-F2.23, GD-F250, and GD-F560) had lower stimulated LGR compared to group GD. The phthalocyanine at the lowest dose (F2.23) yielded the lowest stimulated LGR.

Basal LPR did not differ (P>0.05) between groups GD and GC. Groups GD-G and GD-F2.23 showed higher basal LPR compared with group GD. Groups GD-F250 and GD-F560 decreased basal LPR in comparison with group GD-G. Of the three doses of phthalocyanines, F250 caused the greatest decrease of basal LPR.

During the stimulated (gluconeogenic) perfusion, LPR was higher in group GD than in group GC. Groups GD-F2.23, GD-F250, and GD-F560 had lower LPR during this period compared with group GD, while group GD-G did not differ from group GD (P>0.05).

### **Biochemical determinations**

As reported in Table 2, group GD had GGT, ALP, and FRU significantly higher than group GC, but AST and ALT were not different (P>0.05). All the diabetic groups, given either the hydrogel (GD-G) or hydrogel+phthalocyanine (GD-F), had increased AST compared with group GD (+100 to +120%). In group GD-F250, AST was four times higher; this was also the only group in which ALT was higher than in the other diabetic groups (2 to 2.5 times), as well as had higher GGT and FRU than groups GD, GD-G, and GD-F2.23. The other groups were similar to each other and to group GD (P>0.05).

**Table 2:** Plasmatic markers of liver injury in mice from groups GC, GD, GD-G, GD-F2.23, GD-F250, and GD-F560.

(mg/dL)	GC	GD	GD-G	GD-F2.23	GD-F250	GD-F560
AST	55.00±9.32	60.33±10.93	132.5±7.14b	127.3±22.39	238.2±9.26bcd	143.7±7.91b
ALT	23.88±2.48	24.83±3.19	26.6±1.52	30.17±3.66	64.17±2.14bcd	24.00±3.23
GGT	2.38±1.06	6.50±0.93a	6.25±1.04	5.25±0.71	9.25±1.39bcd	5.22±0.67
ALP	151.6±5.53	306.9±19.84a	317.8±11.37	312.6±8.40	355.6±16.87bcd	190.2±7.46bcd
FRU	0.76±0.05	1.98±0.10a	1.05±0.05b	1.84±0.05bc	1.64±0.05bcd	1.68±0.10bcd

GC: control group; GD: diabetic animals; GD-G: diabetic animals that were injected with hydrogel only; GD-F2.23: diabetic animals that were injected with hydrogel + 2.23 µg/mL phthalocyanine; GD-F250: diabetic animals that were injected with hydrogel + 250 µg/mL phthalocyanine; GD-F560: diabetic animals that were injected with hydrogel + 560 µg/mL phthalocyanine. AST: aspartate amino transferase; ALT: alanine amino transferase; GGT: gamma glutamyl transferase; ALP: alkaline phosphatase; FRU: fructosamine. Data are reported as means±SD; n=7-8/group. a: P<0.05 *vs* GC (*t*-test); b: P<0.05 *vs* GD; c: *vs* GD-G; d: P<0.05 *vs* GD-F2.23 (one-way ANOVA/Tukey).

Among the diabetic animals given subcutaneous injections, group GD-F250 had the highest ALP, while group GD-F560 had the lowest; groups GD, GD-G, and GD-F2.23 did not differ from each other on ALP levels (P>0.05).

The plasmatic levels of FRU were lower in the injected groups compared with group GD; group GD-G had the most marked decrease (-47%). Of the three doses of phthalocyanine, F2.23 showed the smallest reduction of FRU (-7%) compared to group GD.

Except for FRU, the values of the markers from group GD-F2.23 were not different from those of the diabetic group (GD, P>0.05).

Table 3 shows the plasmatic values for GLU, TGL, and CHOL of the experimental groups. As expected for diabetic animals, group GD had higher fasting glucose, TGL, and CHOL compared to group GC.

**Table 3:** Plasmatic profile of mice from groups GC, GD, GD-G, GD-F2.23, GD-F250, and GD-F560.

(mg/dL)	GC	GD	GD-G	GD-F2.23	GD-F250	GD-F560
GLU	75.57±3.10	548.0±19.46a	505.9±21.98b	529.8±23.29	568.9±24.69cd	537.9±26.74
TGL	68.17±3.98	172.8±9.50a	107.8±4.49b	78.33±3.20bc	130.6±5.03bcd	117.8±3.87bcd
CHOL	99.61±2.94	140.9±7.91a	103.4±5.99b	84.64±6.31bc	98.30±6.57bd	94.96±3.88b

GC: control group; GD: diabetic animals; GD-G: diabetic animals that were injected with hydrogel only; GD-F2.23: diabetic animals that were injected with hydrogel + 2.23 µg/mL phthalocyanine; GD-F250: diabetic animals that were injected with hydrogel + 250 µg/mL phthalocyanine; GD-F560: diabetic animals that were injected with hydrogel + 560 µg/mL phthalocyanine. GLU: glucose; TGL: triglycerides; CHOL: total cholesterol. Data are reported as means±SD; n=7-8/group. a: P<0.05 *vs* GC (*t*-test); b: P<0.05 *vs* GD; c: *vs* GD-G; d: P<0.05 *vs* GD-F2.23 (one-way ANOVA/Tukey).

Plasma glucose was lower in group GD-G compared with group GD, and group GD-F250 had the highest plasma glucose. Groups GD-F2.23 and GD-F560 did not differ from group GD (P>0.05).

All the groups given hydrogel or hydrogel+phthalocyanine had lower TGL than group GD (GD-G -38%; GD-F2.23 -55%; GD-F250 -25%; GD-F560 -32%), the lowest value being that of group GD-F2.23. These groups also had lower values of CHOL compared with group GD (GD-G -27%; GD-F2.23 -43%; GD-F250 -29%; GD-F560 -36%). Once again, F2.23 caused the lowest value (significantly lower than GD-G and GD-F250).

Based on the plasmatic and hepatic results, the liver glycogen and free glucose contents of groups GC, GD, and GD-F2.23 were determined. The values for liver glycogen content, in mmol glucose/g liver, were as follows: GC = 0.694±0.030; GD = 0.584±0.085; GD-F2.23 = 0.598±0.054. These were not significantly different (P>0.05). Free glucose (in mmol/g liver) was similar (P>0.05) in groups GD (0.046±0.008) and GD-F2.23 (0.044±0.010), but both were higher (P<0.05) than in group GC (0.023±0.009).

### *In vivo* experiments

The dose of hydroxy-aluminum phthalocyanine that had less effect on liver glucose metabolism and on plasmatic markers of liver injury was selected for the *in vivo* assays. Of the three doses tested, F2.23 decreased stimulated LGR, reduced the plasmatic levels of TGL, CHOL, and FRU, and did not change basal LGR and the other plasmatic parameters. Therefore, GTT and ITT were conducted using this dose of phthalocyanine (group GD-F2.23).

**Figure 2:**
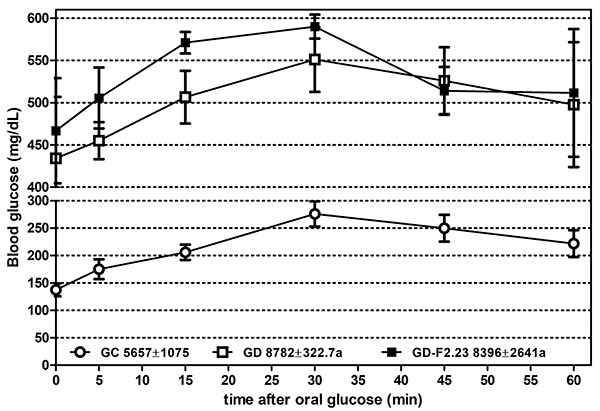
Glucose tolerance test and blood glucose variation of mice from groups GC, GD, and GD-F2.23. GC: control group; GD: diabetic animals; GD-F2.23: diabetic animals that were injected with hydrogel + 2.23 µg/mL phthalocyanine. Data are reported as means±SD; n=4-6/group. a: P<0.05 *vs* GC, one-way ANOVA/Tukey.

Figure 2 shows the blood glucose profile during the GTT and the AUC of glucose variation during the 60 min of the test. Blood glucose was significantly higher in the diabetic groups (GD and GD-F2.23) than in the control (GC) during the whole test. Groups GD and GD-F2.23 had AUC higher than group GC, without interference of phthalocyanine on this parameter (P>0.05 GD *vs* GD-F2.23).

**Figure 3:**
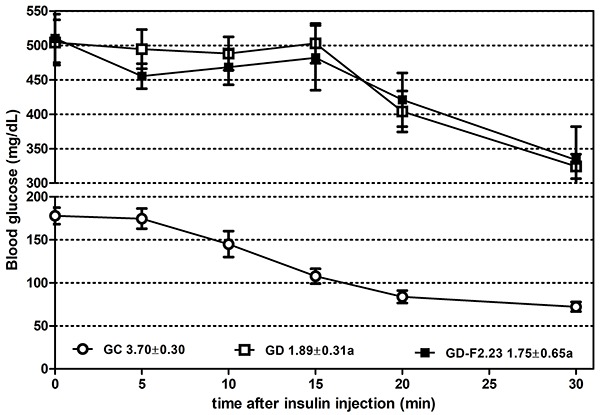
Insulin tolerance test and blood glucose decay index of mice from groups GC, GD, and GD-F2.23. GC: control group; GD: diabetic animals; GD-F2.23: diabetic animals that were injected with hydrogel + 2.23 µg/mL phthalocyanine. Data are reported as means±SD; n=4-6/group. a: P<0.05 *vs* GC, one-way ANOVA/Tukey.

Figure 3 shows the blood glucose profile of the groups during the ITT and blood glucose decay index (kITT). Blood glucose was significantly higher in groups GD and GD-F2.23 during the whole test, while kITT was significantly lower than in group GC. Group GD-F2.23 did not differ from group GD in these parameters (P>0.05).

## Discussion

This investigation tested systemic and hepatic effects of three different doses of the photosensitizer hydroxy-aluminum phthalocyanine and its Pluronic^®^+Carbopol^®^ hydrogel in mice with T1DM not treated with insulin. The treatment of diabetic skin ulcers with PDT has been showing good results (7), but *in vivo* assays employing phthalocyanines in experimental models have not been performed yet.

Initially, the data of the diabetic animals of group GD (hyperglycemia, dyslipidemia, and high basal LGR) showed that diabetes was successfully induced by alloxan and that the model was suitable for the purposes of the investigation, so that the experiments could be confidently performed.

In general terms, the results indicated that both the hydrogel (group GD-G) and the phthalocyanine at any dose (groups GD-F) influenced liver glucose metabolism, markers of liver injury, and plasma glucose and lipids, with no consistent pattern in these modifications, that is, no dose-related progressive increase or decrease. Therefore, the criterion for the choice of the dose of phthalocyanine (F2.23) for the *in vivo* tests was that of no/less/positive influence on the diabetic condition as exhibited by the reference diabetic group (GD).

### Liver perfusion

The liver is the major organ of metabolic homeostasis; its strategic location between the gastrointestinal system and the systemic circulation favors its role on the regulation of many biological processes. Among these are the pathways of blood glucose regulation: glycogen synthesis, glycogenolysis (glycogen degradation), and gluconeogenesis (glucose synthesis from non-carbohydrate substrates) (25).

The liver basal perfusion (without the addition of gluconeogenic precursors) showed higher LGR in all the diabetic groups compared with the control (GC). As during this period there is no glucose precursor, the glucose released comes from that previously stored in the liver as glycogen (26). Therefore, the higher basal LGR may be the result of increased liver glycogenolysis, as glycogen stores could be enhanced because of the long-term hyperglycemia, the predominant feature of non-insulin treated T1DM (27). Although glycogen content in groups GC and GD was similar, the amount of free glucose was higher in the diabetic mice.

The hydrogel increased basal LGR by 1.5 times and LPR by 3 times compared to group GD. These are indicatives that the hydrogel increased glycogenolysis and glycolysis. The relevant point for the purpose of this investigation is that the hydrogel is not a metabolically inert compound, as it modified basal liver energy metabolism.

The addition of phthalocyanine to the hydrogel (groups GD-F) changed the liver responses even further. In the basal period, F2.23 decreased LGR by 33% compared to group GD-G, bringing it to the GD value; however, it maintained basal LPR as high as the hydrogel, that is, about 200% above that of group GD. This could be a direct effect of the phthalocyanine in decreasing glycogenolysis without interfering with the proposed effect of the hydrogel on glycolysis. Groups GD-F250 and GD-F560 decreased basal LPR, indicating that they could be inhibiting glycolysis. In addition, group GD-F560 markedly increased basal LGR (+138% relative to groups GD and GD-F2.23, +54% relative to GD-G and +90% relative to GD-F250). In this way, by associating a supposed inhibition of basal glycolysis with increased glycogenolysis, F560 may contribute dangerously to worsen the hyperglycemic condition of the animal, which was reflected in the fasting glucose of this group, the highest of the diabetic groups.

The infusion of the gluconeogenic precursors lactate and glycerol at saturating concentrations (4 mM) measures the capacity of the gluconeogenic pathway of the liver. In this condition, LGR and LPR of group GD were, respectively, lower and higher than in group GC. Group GD-G did not change these values, showing that the hydrogel did not interfere with gluconeogenesis in the diabetic animal.

On the other hand, the three doses of phthalocyanines decreased stimulated LGR and LPR compared to group GD. In fact, by reducing LGR even more than the hydrogel, phthalocyanines seem to inhibit gluconeogenesis and/or increase the indirect pathway of glycogen synthesis (which is more plausible); however, the decreased pyruvate release may indicate decreased glycolysis. Both effects suggest that phthalocyanines strongly affect the gluconeogenic and glycolytic metabolism of the liver even in the absence of illumination (i.e., not activated by light).

### Plasmatic dosages

When the liver parenchyma is injured, the enzymes AST and ALT are released and their concentrations in the bloodstream increase (28). Plasmatic AST indicates deep cellular injury that reaches the mitochondria, where the enzyme is located; increased plasmatic ALT is a sign of less deep cellular injury, at the level of the plasma membrane, given its cytosolic location. GGT, a very active enzyme in glutathione metabolism (29), can be an early indicator of oxidative stress. ALP is a transport protein of the plasma membrane of the biliary canaliculi and is associated with canalicular injury (30).

In this investigation, diabetes itself (group GD) did not change AST and ALT, but the increased GGT and ALP confirmed the increased oxidative stress typical of T1DM in the liver and showed that, in this experimental model, diabetes damaged the biliary system.

The dosages of the markers of liver injury indicated that both the hydrogel and the phthalocyanines caused deep cellular injury in the hepatic parenchyma (suggested by the increased AST). F250 showed the greatest interference with these markers, increasing not only AST but also GGT (+50%) and ALP (+20%) compared to the other diabetic groups. This suggests greater power of injury on the liver biliary system and increased oxidative stress.

FRU is the glycation product of serum proteins resulting from high blood glucose levels; it has a half-life of 14-21 days (31) and is an indicator of glycemic control during this period. The plasmatic levels of FRU showed that group GD had FRU 150% higher than group GC, which is predictable and confirms the major feature of the diabetic model used - persistent hyperglycemia. Surprisingly, group GD-G decreased FRU by 45%. This suggests that the hydrogel could be decreasing the level of chronic hyperglycemia; nevertheless, fasting glycemia in group GD-G decreased only 10% relative to GD. Phthalocyanines reverted the effect of the hydrogel by increasing FRU to values similar to those of group GD.

As expected for the T1DM model, there was an increase in plasmatic TGL and CHOL. These values were lower in group GD-G, with cholesterol values close to those of non-diabetic mice (group GC). Similarly, all the phthalocyanines decreased these parameters. Thus, both the hydrogel and the phthalocyanines seem to interfere with lipid metabolism in diabetes.

Defects in insulin release, typical of T1DM and the animal model used, imbalance the release of glucagon, corticosteroids, and catecholamines (27,32) and predispose to dyslipidemia and diabetic ketoacidosis. This is because of the increased flux of free fatty acids from peripheral lipolysis, as well as *de novo* lipogenesis, which increases the release of VLDL by the liver, hepatic oxidation of lipids, and ketoacidosis (27,33). The increased total cholesterol in diabetic patients is a relationship between hypertriglyceridemia, oxidation and glycation of LDL particles, and the low levels of HDL-cholesterol (34). As dyslipidemia is a severe complication of diabetic patients, GD-F2.23 had the best effect on these parameters, decreasing TGL and CHOL to values very close to those of non-diabetic controls (GC).

### Glucose tolerance and insulin sensitivity

GTT is characterized by an increase of blood glucose after the oral administration of a glucose load. In non-diabetic animals and patients, blood glucose increase is limited by endogenous insulin release, followed by a quick return to basal values that characterizes glucose tolerance. The initial excessive increase of blood glucose followed by a slow return to basal levels indicates glucose intolerance, resulting in higher AUC of blood glucose variation. This response is typical of a diabetic animal, which has absence of or impaired insulin action and inability to uptake/metabolize the glucose load (35).

On the other hand, insulin resistance is a metabolic derangement typical of individuals having T2DM, untreated T1DM, diabetic ketoacidosis, and obesity (36). ITT was the first direct *in vivo* technique to assess insulin sensitivity. The method consists of a single injection of regular insulin and the evaluation of blood glucose decay during the first 30 min of the test (kITT). This index reflects glucose uptake by the tissues, as well as the inhibition of glucose release by the liver, induced by the injected insulin. The faster and more intense the glucose decrease, the larger the kITT and the insulin sensitivity.

As expected for a T1DM model, group GD had larger blood glucose variation (+52%) and lower kITT (-48%) than the controls (GC). The similar percentages obtained for these two assays indicate that there is an inversely proportional relationship between them, that is, the lower the insulin sensitivity, the greater the glucose intolerance.

Phthalocyanine F2.23 did not change GTT and ITT compared to group GD. This is, to a certain point, a positive observation, because the compound (phthalocyanine) or its vehicle (hydrogel) are not supposed to interfere with *in vivo* glucose homeostasis, which would make glycemic control more difficult and unstable for diabetic patients under insulin therapy.

The effect of F2.23 in decreasing glycogenolysis and keeping basal glycolysis can be beneficial to the diabetic patient, in the sense that these changes do not increase blood glucose to even higher levels than those already existing in T1DM. This did not happen with the other doses of phthalocyanines (F250 and F560), which increased liver glucose release. The results suggest a possible important effect of F2.23 in decreasing liver glucose release that could contribute to decrease blood glucose in T1DM, in addition to its direct effect on the healing of skin ulcers through PDT.

The hydrogel of Pluronic^®^ and Carbopol^®^ was not metabolically inert and the photosensitizer hydroxy-aluminum phthalocyanine, even in the absence of light, changed liver glucose metabolism, plasmatic biochemical parameters, and markers of hepatic injury. Among the doses tested, F2.23 (the lowest dose) did not change several of the tested parameters and promoted some important and apparently beneficial - or at least not aggravating - alterations, for the diabetic condition. The lack of effect of F2.23 on insulin sensitivity and glucose tolerance should also be regarded as positive, once its use on PDT would not create additional metabolic challenges for the diabetic patient.
